# Folic Acid Supplementation Is Suboptimal in a National Cohort of Older Veterans Receiving Low Dose Oral Methotrexate

**DOI:** 10.1371/journal.pone.0168369

**Published:** 2016-12-15

**Authors:** Gabriela Schmajuk, Chris Tonner, Yinghui Miao, Jinoos Yazdany, Jacqueline Gannon, W. John Boscardin, David I. Daikh, Michael A. Steinman

**Affiliations:** 1 Department of Medicine, University of California San Francisco, San Francisco, CA, United States of America; 2 Department of Medicine, Veterans Affairs Medical Center–San Francisco, San Francisco, CA, United States of America; 3 School of Public Health, Yale University, New Haven, CT, United States of America; VU University Medical Center, NETHERLANDS

## Abstract

**Objectives:**

Co-prescription of folic acid in patients receiving low dose oral methotrexate is recommended because it reduces adverse events and prolongs the use of methotrexate (MTX). However, little is known about how often new users of methotrexate are co-prescribed folic acid, and what factors are associated with its use. We aimed to determine the prevalence, predictors of, and persistence of folic acid use in a population-based cohort of MTX users with rheumatic diseases.

**Methods:**

Using a national, administrative database of patients seen through the Veterans Health Administration (VHA) that included pharmacy and laboratory data, we performed an observational cohort study of veterans over 65 years old who were new users of MTX. We used log-binomial regression to identify independent predictors of folic acid use and Kaplan Meyer survival analysis to examine persistence of folic acid over time.

**Results:**

We studied 2467 incident users of MTX. 27% of patients were not prescribed folic acid through the VHA pharmacy within 30 days of MTX initiation. Patients who did not see a rheumatologist were 23% less likely to receive folic acid compared to patients who did have a rheumatologist visit during the baseline period (RR (95% CI) 0.77 (0.72, 0.82). These results remained unchanged even after adjusting for demographic, clinical, and other factors (adjusted RR (95% CI) 0.78 (0.74, 0.85)). After 20 months, only 50% of patients continued to receive folic acid.

**Conclusions:**

In a nationwide VHA cohort of new users of oral MTX, many patients did not receive folic acid or discontinued it over time. Rheumatologists were more likely to prescribe folic acid than other providers. These data highlight the need to improve patient safety for users of methotrexate by standardizing workflows for folic acid supplementation.

## Introduction

Low-dose oral methotrexate (MTX) is the first-line treatment for rheumatoid arthritis (RA), psoriatic arthritis, and other systemic autoimmune diseases, which affect an estimated 3 million people in the U.S.[1,2] Although methotrexate is effective and inexpensive, side effects limit its use in many patients. In particular, patients discontinue methotrexate because of nausea or vomiting, hair loss, and liver function test elevations more often than for lack of efficacy.[3,4] Multiple studies–including a randomized controlled trial–have demonstrated the benefits of folic acid supplementation in reducing the incidence of liver function test abnormalities and prolonging the time to discontinuation of methotrexate, with no reduction in its effectiveness.[5,6] Professional society guidelines of the American College of Rheumatology (ACR) and the European League Against Rheumatism (EULAR) advocate the use of folic acid among all patients receiving methotrexate.[7,8]

Despite the strong evidence supporting its use, few studies have reported on practice patterns around folic acid supplementation or predictors of its use.[9,10] Prior studies focused on prevalent users of methotrexate, which may have biased their results, since longstanding users of a drug may be more adherent and/or more tolerant of it.[11,12,13] Furthermore, only one study[9] reported on patients under the care of non-rheumatologists (such as primary care physicians or dermatologists, many of whom prescribe MTX).

We sought to determine the frequency and predictors of folic acid supplementation among MTX users in a national cohort of older adult patients cared for in the United States Department of Veterans Affairs healthcare system, the largest integrated health system in the U.S. We chose to focus on older adults because they are a large and growing segment of the population of patients with rheumatic diseases who are at particular risk for medication side effects.[14]

## Methods

This project was approved by University of California–San Francisco and San Francisco Veterans Affairs Medical Center internal review boards; waiver of informed consent was granted due to the nature of the study.

### Data sources

The national Veterans Health Administration (VHA) databases contain information on patient demographics (age, sex, race) as well as inpatient and outpatient claims that provide information on specific medical conditions, number and types of healthcare encounters, pharmacy prescriptions (including dose and days’ supply for medications supplied through VHA pharmacies) and information on laboratory tests performed through the VHA, including test date, test performed, and test result. The VHA also permits linkage to national Medicare databases. We had access to data from fiscal years 2001–2012.

### Study population

We performed a retrospective cohort study of incident users of methotrexate. We included subjects who received a new prescription for MTX of at least 28 days between March 1, 2001 and July 30, 2012. The date of the first MTX prescription was designated as the index date. Patients were included if they had one or more VHA medical encounters during the 365 days prior to the index date and one or more VHA medical encounters or laboratory result during the 90 days after the index date. Patients were also required to have at least 180 days of methotrexate-free use of the VHA healthcare system prior to the index date. The last possible index date was July 1, 2012, in order that all patients have at least 90 days of follow-up.

As described in our prior studies of MTX users, a significant fraction of older veterans receive care outside the VHA through a concurrent Medicare benefit.[5,15] In order to ensure that we fully captured all pharmacy prescriptions and laboratory outcomes in our VHA data, we excluded patients who may have obtained prescriptions or laboratory tests through Medicare. We did this by removing all subjects with any medical encounter (inpatient or outpatient) billed to Medicare during the 365 days prior to the index date or ≥ 1 Medicare laboratory testing claim between the index date and the end of the follow-up period. In addition, subjects were excluded if they carried a diagnosis of inflammatory bowel disease (ICD-9 codes 555.x or 556.x) because these patients may be given routine folic acid supplements unrelated to MTX use. [16]

### Outcome

The main outcome was the receipt of folic acid at the time of the first MTX prescription. In order to account for variations in practice, we allowed folic acid dispensation to occur 30 days before or 30 days after the index MTX prescription through the VHA pharmacy. Subjects were categorized as users of folic acid if they received at least a 28-day supply of stand-alone folic acid (at any dose) from a VHA pharmacy during this period. We also assessed patients for use of possible folic acid equivalents, including folinic acid or leucovorin: Five patients received leucovorin within 30 days of MTX start, and of these, 3 also started folic acid during this time; the 2 other leucovorin users were considered non-users of folic acid. Multivitamins were not considered acceptable sources of folic acid because they generally provide only 400 mcg of folic acid.

In a second analysis, we followed patients who received folic acid within 30 days of their first MTX prescription over time to assess the rate of folic acid discontinuation. Discontinuation was defined as going 60 days without supply of folic acid through the VHA pharmacy. [17]

### Covariates

We identified patient and physician factors associated with the receipt of folic acid within 30 days after the index date.

#### Comorbid conditions

Comorbid conditions were assessed during the 365 days leading up to the index prescription. To identify comorbidities, we reviewed all outpatient encounter diagnoses and inpatient discharge diagnoses. Conditions were considered present if they were encoded ≥ 2 times as an outpatient claim or ≥ 1 time as an inpatient claim during this period; these methods have been shown to have good sensitivity and specificity for identifying common diagnoses in VHA administrative data.[18,19] Comorbid conditions of interest included the primary diagnosis requiring MTX (see below) and a modified Charlson score was calculated according to the Deyo protocol.[20]

#### Primary diagnosis requiring methotrexate

The primary diagnosis requiring methotrexate was assessed during the 365 days leading up to the index prescription until 30 days after the index date. If a patient had 2 or more diagnosis codes for rheumatoid arthritis or psoriatic arthritis, this was assumed to be the primary diagnosis requiring methotrexate. If patients had both diagnoses, they were assumed to have psoriatic arthritis;[21] if they had either just 1 diagnosis for rheumatoid arthritis or psoriatic arthritis, or neither diagnosis, they were placed into the “other” category.

#### Concomitant medication use

Data from VHA’s outpatient pharmacy were used to tabulate the number of unique medications used by each patient during the 365 days leading up to the index prescription. Subjects were categorized as users or non-users of the following groups of medications if they had at least a 28-day supply of any of the included drugs during the baseline period. Disease modifying agents were categorized as biologic (including those available by 2012: adalimumab, anakinra, etanercept) or non-biologic (azathioprine, cyclophosphamide, cyclosporine, gold, hydroxychloroquine, leflunomide, minocycline, penicillamine, or sulfasalazine). Of note, intravenous-infused biologics were not included here because they were not captured by the outpatient pharmacy database. Glucocorticoids included oral prednisone, methylprednisolone, prednisolone, or dexamethasone.

#### Health care utilization and provider types

The number of face-to-face visits to primary care physicians or subspecialists during the 365 days prior to the index date was tallied to give an estimate of health care utilization. We also evaluated which specific provider types (rheumatologist, dermatologist) participated in the care of the patient from 365 days prior until 5 days after the index date. Exposure to particular provider types was determined via a “clinic visit type” code.

#### Baseline laboratory results

Data from the VHA’s laboratory files were used to determine patient pre-MTX renal function (based on creatinine and calculated estimated GFR) and pre-MTX AST or ALT levels (dichotomized as normal or above upper limit of normal, using the upper limits of normal at our institution (AST ULN 35 mg/mL; ALT ULN 60 mg/mL).

### Statistical analysis

Baseline characteristics were compared between patients who did and did not receive folic acid. Relative risk estimation by log-binomial regression was used to calculate unadjusted and adjusted relative risks of initial folic acid prescription among different subgroups. Variables included in the multivariate models were selected a priori, dichotomized, and tested for collinearity.

In an analysis of folic acid discontinuation, we followed the subset of patients who initially received folic acid over time. We used a Kaplan-Meier estimate of the survival distribution to examine the rate of discontinuation of folic acid. Patients were censored at time of death or when patients went > 60 days without a supply of folic acid. Non-censored patients were followed until the end of exposure to MTX (end of days supply + 60 days).

All analyses were performed using SAS version 9.3 (SAS Institute Inc.).

## Results

We included 2467 new users of methotrexate who were followed for a mean (SD) of 428 (531) days. Twenty-seven percent were not prescribed folic acid within 30 days of the first methotrexate prescription. Baseline characteristics are described in Table 1. The majority of patients were male and Caucasian. The mean starting dose for methotrexate was 12.8 mg (SD 5.3, range 1.2–40.0 mg) weekly. The majority carried a diagnosis of rheumatoid arthritis. The most common diagnoses in the “other” category included polymyalgia rheumatica and vasculitis.

**Table 1:** Baseline characteristics of incident methotrexate users.

Characteristics		
N		2467
Demographics, N (%)		
	Age at cohort entry, mean (SD)	72.0 (7.0)
	Male	2364 (97)
	Non-white race	296 (12)
Methotrexate mean weekly dose, mg (mean SD)		12.8 (5.3)
Primary diagnosis requiring methotrexate, N (%)		
	Rheumatoid arthritis	1413 (57)
	Psoriasis/psoriatic arthritis	491 (20)
	Other	563 (23)
Comorbidities/baseline labs, N (%)		
	Charlson score (mean, SD)	1.8 (1.8)
	Poor renal function (< 60 by EGFR)*	691 (34)
	Baseline AST or ALT abnormal**	162 (7)
Baseline medication use, N (%)		
	Non-biologic DMARD	502 (20)
	Biologic DMARD	59 (2)
	Glucocorticoid	1205 (49)
Health care access during 12 months prior– 5 days after index methotrexate prescription, N (%)		
	# Outpatient encounters, mean (SD)	29.7 (27.9)
	# Primary care or subspecialty visits, mean (SD)	6.2 (6.4)
	# Outpatient medications, mean (SD)	10.4 (6.9)
	Any rheumatology visit	1612 (65)
	Any dermatology visit	679 (28)

* N = 2026

** N = 2283; abnormal defined as > upper limit of normal; for AST > 35 mg/mL; for ALT > 60 mg/ml.

To determine independent factors associated with folic acid receipt, we calculated unadjusted and adjusted relative risks for folic acid receipt and baseline characteristics (Table 2). In the unadjusted analysis, we found an RR (95% CI) of 0.77 (0.72, 0.82) for patients who did not see a rheumatologist compared to those who did during the baseline period: in other words, patients who did not see a rheumatologist were 23% less likely to receive folic acid. These results remained unchanged even after adjusting for demographic, clinical, and other factors (adjusted RR (95% CI) 0.78 (0.74, 0.85); model adjusts for all variables shown in Table 2). Older patients were also slightly less likely to receive folic acid. Other demographic and clinical factors were not significant in the adjusted model. In an unadjusted analysis, we found an RR 1.02 (0.96, 1.07)) for folic acid use for patients who did not see a dermatologist compared to those who did during the baseline period.

**Table 2:** Factors associated with folic acid receipt, and multivariate model predicting folic acid receipt within 30 days of first methotrexate prescription.

Characteristics	Not prescribed folic acid	Prescribed folic acid N (%)	Unadjusted RR	Adjusted RR†
	N (%)			
Male (vs. Female)	647 (96)	1741 (97)	1.09 (0.83, 1.43)	1.12 (0.96, 1.30)
Non-White Race (vs. White)	64 (10)	232 (13)	**1.09 (1.02, 1.16)**	1.01 (0.95, 1.08)
Age > 70 (vs. < = 70)	429 (64)	1054 (59)	**0.93 (0.88, 0.98)**	**0.94 (0.90, 0.99)**
Methotrexate dose > 15 mg/week (vs. < = 15)	209 (31)	559 (31)	1.00 (0.95, 1.05)	1.01 (0.96, 1.06)
Rheumatoid arthritis as diagnosis requiring MTX (vs. all others)	346 (51)	1067 (59)	**1.09 (1.04, 1.15)**	1.01 (0.96, 1.07)
Charlson score > 3 (vs. < = 3)	94 (14)	272 (15)	1.03 (0.96, 1.10)	0.99 (0.93, 1.05)
Abnormal transaminases prior to MTX start (vs. Normal)*	50 (8)	112 (7)	0.93 (0.84, 1.04)	0.96 (0.87, 1.05)
Glucocorticoid use at baseline (vs. none)	273 (41)	932 (52)	**1.11 (1.06, 1.16)**	1.02 (0.97, 1.07)
Number of outpatient encounters > 14 (vs. < = 14)	374 (56)	1200 (67)	**1.12 (1.06, 1.18)**	1.05 (0.99, 1.11
Number of outpatient medications > 10 (vs. < = 10)	242 (36)	840 (47)	**1.11 (1.05, 1.16)**	1.05 (1.00, 1.11)
No rheumatologist visit (vs. Yes rheumatologist visit)	340 (51)	515 (29)	**0.77 (0.72, 0.82)**	**0.78 (0.74, 0.85)**

* N = 2283; abnormal defined as > upper limit of normal; for AST > 35 mg/mL; for ALT > 60 mg/ml.

† Adjusted RRs from a multivariate model that adjusted for all variables listed in this table (and no others).

**Fig 1:**
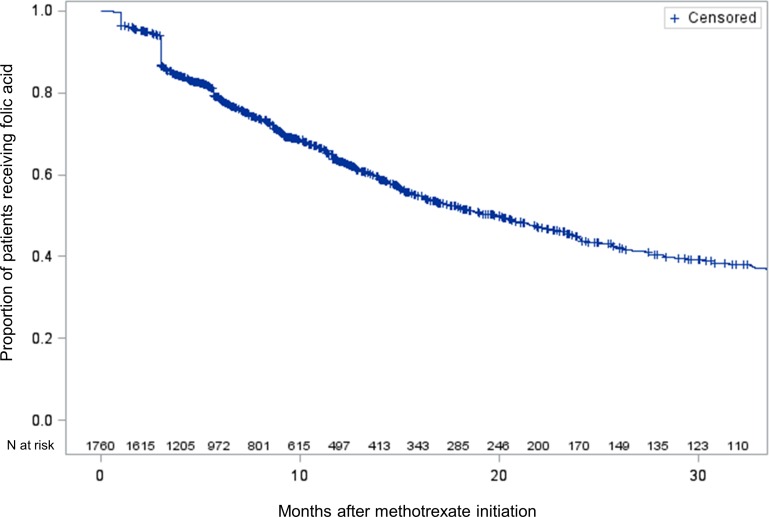
Patient who received folic acid from the VHA pharmacy were followed over time to observe discontinuation rates. Patients were censored at time of death or when patients went > 60 days without a supply of folic acid. Non-censored patients were followed until the end of exposure to MTX (end of days supply + 60 days).

Finally, we followed patients who received folic acid up to 30 days after the time of their first prescription for MTX (N = 1760). (We excluded 34 patients who only received folic acid in the 30 days prior to their index MTX prescription.) Two steep declines occur after 1 month and 3 months, likely related to the number of refills provided on the initial prescription. After 20 months, only 50% of patients continued to receive folic acid (Fig 1).

## Discussion

In this cohort of elderly patients who were new users of methotrexate, folic acid supplementation was suboptimal. We found that more than a quarter of older patients starting low-dose methotrexate did not receive folic acid through VHA pharmacies, and that only 50% of patients continued to receive folic acid after 20 months of receiving methotrexate. Patients who saw a rheumatologist were more likely to receive folic acid with the first MTX prescription, although even in this group, folic acid supplementation was lower than expected (77%). These findings highlight the need to develop strategies to ensure universal folic acid supplementation for methotrexate users. Two prior studies have examined folic acid supplementation among prevalent methotrexate users. One study assessed prevalent MTX users seen by a mix of providers through the National Ambulatory Care Survey (NAMCS).[9] They reported that in 2009, 45% of MTX users received folic acid supplementation. Although medication use is reported by physicians in NAMCS, only a limited number of medications are captured on a given visit form, so this may be an underestimate of folic acid use in this population. In a study of patients in the CORRONNA registry, authors reported a much higher proportion (76%) of prevalent MTX users receiving folic acid, although all patients were followed by rheumatologists.[10] The fraction of patients receiving folic acid in CORRONNA is similar to that found in our study, despite the differences in populations.

Our study is unique because it examined an incident-user cohort of methotrexate users. Prior studies that included prevalent MTX users may have been enriched for adherent patients or those tolerant to MTX. These studies therefore could have been subject to significant selection bias.[11,12,13] Our new-user design allowed us to study patients during a critical period to address patient safety for a potentially toxic drug.[22] Additionally, because our sample was not clinic-based, we were able to examine rates of folic acid receipt among patients seen by a variety of providers. Interestingly, baseline sociodemographic and patient clinical factors, such as biologic disease modifying agent use, and history of LFT elevations were not associated with folic acid prescription, in spite of them conferring an increased risk for hepatotoxicity.[5] Instead, we found that the strongest predictor of appropriate care was contact with a rheumatologist, which is consistent with trends around other measures of quality of care in patients with rheumatic conditions.[23,24]

This study has several limitations: As with most studies of cohorts derived from the Veterans Health Administration, most patients were male;[25] we also focused on adults age 65 and older. Although this may limit the generalizability of our findings, our data are nationally representative and capture a population at high risk for adverse drug events. It is possible that some of the included individuals may have been misclassified as new users of MTX; however, most VHA pharmacies permit a maximum 90-day fill for MTX, so it is likely that our 180-day wash out period effectively identified incident users.[11] Some patients may have been prescribed folic acid, but may have never received it from the pharmacy, and therefore should have been characterized as non-users. However, given that we defined our cohort based on having received an initial fill of a concomitant medication (MTX), this is less likely. Perhaps most importantly, folic acid supplements can be obtained over-the-counter, and we were concerned that patients may have been misclassified as non-users when in truth they were obtaining folic acid supplements outside the VHA pharmacy, either from other physicians or by purchasing supplements themselves. We attempted to mitigate this by excluding patients with any Medicare claims, thus restricting our sample to patients who were receiving care exclusively through the VHA and thus were most likely to receive all of their medications through the VHA pharmacy. Although patients were at liberty to buy folic acid supplements from over the counter sources, a quality improvement study performed at the San Francisco VA hospital showed that only 20% patients without a folic acid prescription in the VHA electronic health record had over the counter folic acid use recorded in the chart (unpublished data). Applying this information to our study, even if a quarter of patients without folic acid prescribed were misclassified, rates of folic acid prescription would still hover near 80%. All together, the evidence presented here suggests a gap in folic acid supplementation for methotrexate users in the VHA.

The suboptimal rates of folic acid supplementation among methotrexate users identified in this study have implications for patient care and safety. Folic acid supplementation can reduce side effects that limit therapeutic dose escalations of methotrexate for conditions like rheumatoid arthritis and can prevent medication side effects such as MTX-associated LFT elevations. Policy makers should strive to improve folic acid coprescription on first receipt of methotrexate and thereafter.[26] Providers, including pharmacists, and patients should be educated about its potential benefits. A recent study of a diverse cohort of RA patients demonstrated that only 64% of MTX users were aware of the role of folic acid when used with methotrexate.[27] Providers can be incentivized to provide folic acid prescriptions and patient education through the use of quality measures and feedback on measure performance.[28] Healthcare systems and insurance companies can develop drug coverage policies that promote folic acid use by eliminating copayments for folic acid supplements, as has been demonstrated in the case of statins.[29] Clinical decision support tools could prompt providers at the point of care to prescribe folic acid along with MTX. Electronic health records or pharmacy systems could be designed to automatically “bundle” methotrexate and folic acid prescriptions, as has been successfully demonstrated with calcium/vitamin D and prednisone use in the VHA setting.[30]

In summary, despite strong evidence that folic acid supplementation should be universal among MTX users, many older, new users of low-dose oral MTX did not receive a prescription for folic acid. Improving folic acid prescription has the potential to significantly reduce the incidence of LFT abnormalities and may allow more widespread and persistent use of MTX, an effective and inexpensive medication for RA and other rheumatic diseases.
